# Regulatory Architecture of the Neuronal *Cacng2/Tarpγ2* Gene Promoter: Multiple Repressive Domains, a Polymorphic Regulatory Short Tandem Repeat, and Bidirectional Organization with Co-regulated lncRNAs

**DOI:** 10.1007/s12031-018-1208-x

**Published:** 2018-11-26

**Authors:** B.P.A. Corney, C.L. Widnall, D.J. Rees, J.S. Davies, V. Crunelli, D.A. Carter

**Affiliations:** 1 School of Biosciences, Cardiff University, CF103AX, Cardiff, UK; 2 Molecular Neurobiology, Institute of Life Science, Swansea University, Swansea, SA2 8PP UK

**Keywords:** Synaptic, AMPA, TARP, REST, CaRF, lncRNA, Bidirectional, Absence epilepsy

## Abstract

CACNG2 (TARPγ2, Stargazin) is a multi-functional regulator of excitatory neurotransmission and has been implicated in the pathological processes of several brain diseases. *Cacng2* function is dependent upon expression level, but currently, little is known about the molecular mechanisms that control expression of this gene. To address this deficit and investigate disease-related gene variants, we have cloned and characterized the rat *Cacng2* promoter and have defined three major features: (i) multiple repressive domains that include an array of RE-1 silencing transcription factor (REST) elements, and a calcium regulatory element-binding factor (CaRF) element, (ii) a (poly-GA) short tandem repeat (STR), and (iii) bidirectional organization with expressed lncRNAs. Functional activity of the promoter was demonstrated in transfected neuronal cell lines (HT22 and PC12), but although selective removal of REST and CaRF domains was shown to enhance promoter-driven transcription, the enhanced *Cacng2* promoter constructs were still about fivefold weaker than a comparable rat *Synapsin-1* promoter sequence. Direct evidence of REST activity at the *Cacng2* promoter was obtained through co-transfection with an established dominant-negative REST (DNR) construct. Investigation of the GA-repeat STR revealed polymorphism across both animal strains and species, and size variation was also observed in absence epilepsy disease model cohorts (Genetic Absence Epilepsy Rats, Strasbourg [GAERS] and non-epileptic control [NEC] rats). These data provide evidence of a genotype (STR)-phenotype correlation that may be unique with respect to proximal gene regulatory sequence in the demonstrated absence of other promoter, or 3′ UTR variants in GAERS rats. However, although transcriptional regulatory activity of the STR was demonstrated in further transfection studies, we did not find a GAERS vs. NEC difference, indicating that this specific STR length variation may only be relevant in the context of other (*Cacna1h* and *Kcnk9*) gene variants in this disease model. Additional studies revealed further (bidirectional) complexity at the *Cacng2* promoter, and we identified novel, co-regulated, antisense rat lncRNAs that are paired with *Cacng2* mRNA. These studies have provided novel insights into the organization of a synaptic protein gene promoter, describing multiple repressive and modulatory domains that can mediate diverse regulatory inputs.

**Electronic supplementary material:**

The online version of this article (10.1007/s12031-018-1208-x) contains supplementary material, which is available to authorized users.

## Introduction

CACNG2 (TARPγ2, Stargazin*)* is a plasma membrane protein that regulates excitatory neurotransmission in the brain. This 323 amino-acid (in human) protein is an auxiliary subunit of AMPA (α-amino-3-hydroxy-5-methyl-4-isoxazolepropionic acid) glutamate receptors, a so-called trans-membrane AMPAR regulatory protein (TARP) that regulates receptor function and distribution at the synapse (Chen et al. 2000; Jackson and Nicoll 2011). Although a number of different TARPs are expressed in the CNS, indicating some functional redundancy, studies have shown that CACNG2/TARPγ2 does have specific actions, for example, in maintaining AMPAR density in hippocampal synapses (Yamasaki et al. 2016). Of relevance to the current study of *Cacng2* gene expression, experiments have shown that CACNG2 “dose-dependently” affects AMPA receptor gating (Milstein et al. 2007), indicating that expression level could, potentially, be a regulated aspect of CACNG2 function in the brain. Supporting this idea, CACNG2 associates with the (neuronal) activity-regulated cytoskeleton-associated protein, ARC (Zhang et al. 2015), and changes in *Cacng2* expression are correlated with experience-dependent plasticity (Lee et al. 2016; Louros et al. 2014). There is also evidence of both developmental- (Menuz et al. 2009) and disease-related changes in *Cacng2* expression, the latter including several different psychiatric disorders (Beneyto and Meador-Woodruff 2008; Silberberg et al. 2008), and Alzheimer’s disease (Savas et al. 2017).

Given the role of *Cacng2* in synaptic transmission, and this evidence for function/dysfunction-related expression level, it is surprising that very little is known about the molecular mechanisms that regulate *Cacng2* gene expression, including, for example, gene promoter structure. Disease-associated genetic variants are often found in gene regulatory sequences, and, in fact, there is evidence of bipolar disorder-associated variants within putative human *Cacng2* regulatory sequence (Ament et al. 2015). Hence, there is a strong justification for experimental analysis of the regulatory DNA sequences that control *Cacng2* expression.

Our interest in the regulation of this gene is based on studies of absence epilepsy models (Cope et al. 2009; Holter et al. 2005a, 2005b; McCafferty et al. 2018). *Cacng2* is well known as the affected gene in *stargazer* mutant mice (Noebels et al. 1990), but we have primarily studied another genetic model (GAERS; genetic absence epilepsy rats, Strasbourg) that is a polygenic rat model of absence epilepsy (see Cope et al. 2009). The genetic basis of spontaneous spike-and-wave discharges (SWDs) in GAERS is not fully understood; known mutations in *Cacna1h* (Powell et al. 2009) and *Kcnk9* (Holter et al. 2005a) may contribute, but it is likely that additional DNA sequence variants remain to be discovered. One study (Rudolf et al. 2004) has mapped possible mutation sites in a quantitative trait locus (QTL) analysis. One QTL region on rat chromosome 7 is intriguing because it contains the *Cacng2* gene. Although we have shown that the *Cacng2* mRNA sequence is not altered in GAERS rats (Cope et al. 2009), other studies have demonstrated raised levels of *Cacng2* mRNA and protein in GAERS (Powell et al. 2009). These results are consistent with the hypothesis that regulatory DNA sequences controlling *Cacng2* expression could be affected in GAERS.

As noted above*, Cacng2* gene promoter/enhancer sequences have not been directly investigated, and are also of general interest in the molecular neuroscience field because these sequences must contribute to the highly brain-specific expression of this gene (Fukaya et al. 2005; GTEx Consortium 2015). A genome-wide study of the calcium regulatory element-binding factor (CaRF; Pfenning et al. 2010) has identified the *Cacng2* mRNA as one (negatively regulated) CaRF target. In the latter study, a CaRF-associated, CaRE1 element upstream of Cacng2 was also identified (Pfenning et al. 2010), although the functional activity of this site has not been experimentally confirmed. Other than this rather limited data, nothing else is known about the *Cacng2* promoter, and so preliminary cloning and sequencing work is required to define *Cacng2* promoter DNA sequence, and, at the same time, investigate the sequence context of the CaRE site that is conserved across rat, mouse, and human genomes. By conducting this promoter analysis with rat genomic sequence, it will then be possible to investigate possible mutations within GAERS sequence, and consider their functional relevance.

## Methods

### Animals

Rodents were used in accordance with both the UK Animals (Scientific Procedures) 1986 Act of Parliament and Cardiff University ethical review. GAERS and non-epileptic control (NEC) strains were maintained as described (Cope et al. 2009). The health status of the animals was monitored in accordance with these regulations and a veterinarian was consulted if required. Animals were maintained in standard laboratory cages and with standard conditions (14:10 light:dark cycle, lights on: 05.00 h; ad libitum access to food and water) and killed by a Schedule 1 method. Tissues were rapidly dissected, and then either stored briefly on dry ice prior to RNA extraction, or used directly for DNA extraction.

### PCR Analysis of DNA and RNA

Total cellular RNA was extracted from either rat brain samples or cell culture extracts using Trizol (Invitrogen protocol, Thermo Fisher Scientific, Waltham, MA, USA). Rat genomic sequence was amplified from initial rat brain RNA extracts which contain a sufficient background of intact genomic DNA. Mouse genomic DNA was amplified from Maxwell kit-purified mouse tissue (Promega protocol; Promega, Madison, WI, USA). Where cDNA was required from either rat brain or cell culture samples (see below), RNA extracts were DNaseI-purified (Promega protocol), and cDNA was generated with the Superscript II protocol (Life Technologies, Thermo Fisher Scientific) using an Oligo (dT) primer. PCR was conducted using standard procedures with either REDTaq ReadyMix (Sigma, Aldridge, St.Louis, MO, USA) or Q5 Hot-Start High-Fidelity DNA polymerase (NEB, Ipswich, MA, USA). Oligonucleotides used for amplification are listed in Table S1. Amplified products in end-point PCR analysis were visualized after agarose gel electrophoresis, with reference to a DNA ladder (Hyperladder I, Bioline, London, UK or 1 kb ladder, Promega), using GeneSnap (Syngene, Frederick, MD, USA). For cloning and sequence analysis, PCR products were purified (Qiaex II gel extraction kit, Qiagen, Hilden, Germany) and either directly ligated into pGEM-T (Promega protocol;) or, in the case of Q5-amplified products, “A-tailed” (Promega protocol) prior to ligation. Ligations were transformed into JM109 cells (Promega), and transformants were selected for plasmid purification (Wizard SV Miniprep protocol, Promega). PCR products were then sequenced (Eurofins MWG Operon, Ebersberg, Germany). For QPCR analysis, the qPCRBIO SyGreen mix (PCR Biosystems protocol, PCR Biosystems Ltd., London, UK) was used with the Mx3000P system (Agilent, Santa Clara, CA, USA), using the 2^-ΔΔCT^ method for quantitation. In these experiments, where samples of RNA extracted from mouse HT22 cells were analyzed, mouse-specific PCR primers were used, with mActb as the normalization gene (Table S1).

### Expression of *Cacng2* in Cell Culture

**Fig. 1:**
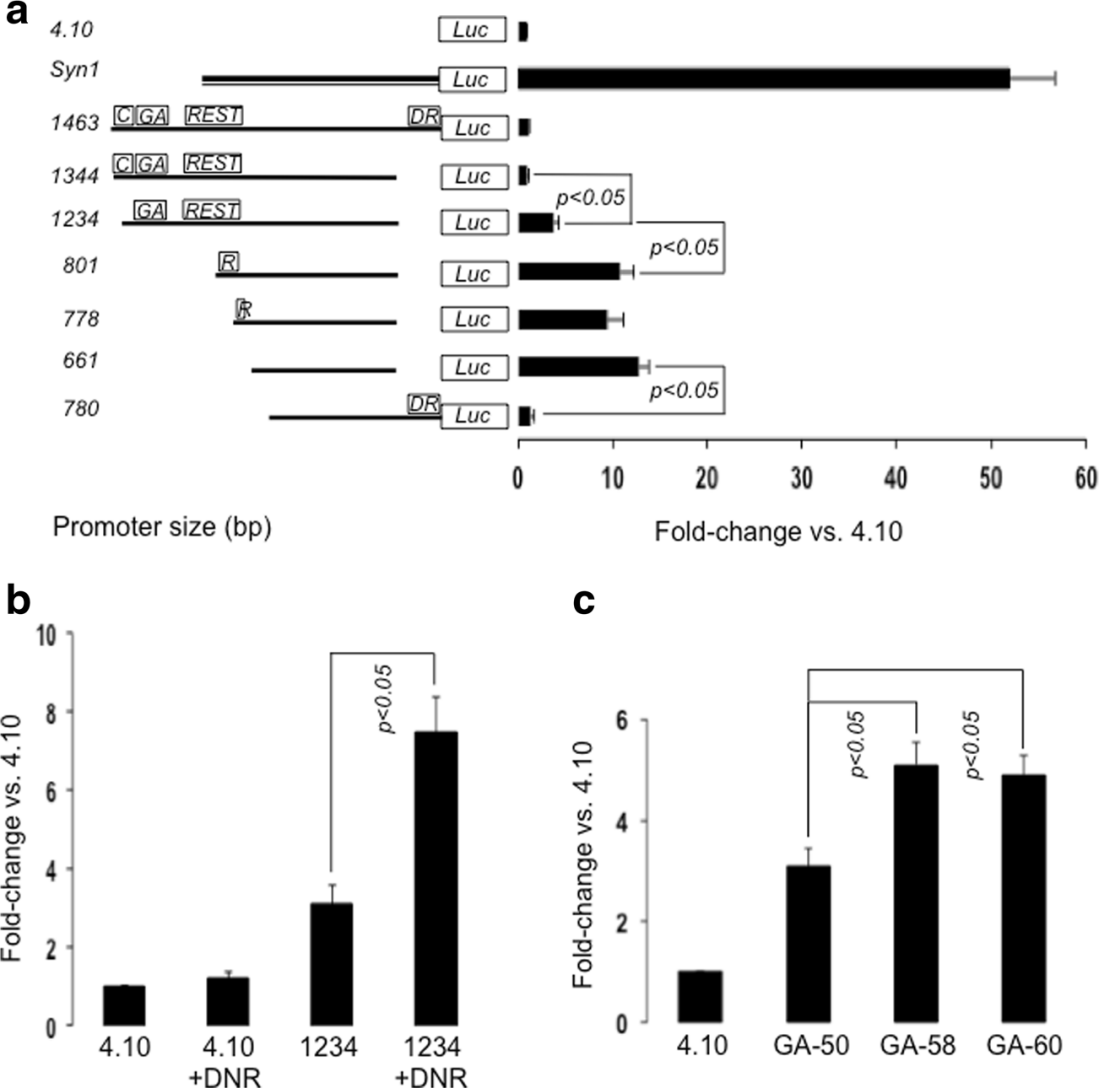
Functional analysis of *Cacng2* promoter activity in transfected cells reveals multiple regulatory sequences, including an array of REST elements and a GA-repeat STR. *Cacng2* sequences were cloned within pGL4.10, transfected into HT22 cells and levels of expression (fold-change relative to empty pGL4.10) were determined by luciferase (Luc) assays. In each experiment, *p* < 0.05 indicates statistically significant differences between different groups, as determined by ANOVA and post hoc analysis. **a** Expression levels of seven different *Cacng2* constructs (mean ± S.E., *n* = 6/group). The relative position of the different elements/regions are indicated by text boxes. C, calcium regulatory element-binding factor consensus site (CaRE); DR, downstream repressive region; GA, GA-repeat STR sequence; REST, array of four REST consensus sites; R fully-boxed, single remaining REST site; R incompletely boxed, partial REST site. For comparison, representative activity of a similar rat *SynI* promoter construct is also shown. This construct served as a positive control in all experiments. **b** Co-transfection of a dominant-negative REST construct (DNR) enhances activity of the 1234 bp *Cacng2* promoter construct. The data are expression levels of constructs (mean ± S.E., *n* = 6/group). *p* < 0.05 indicates a statistically significant difference in *Cacng2* construct activity in the presence of DNR. **c***Cacng2* GA-repeat length affects promoter activity. Expression levels of three different *Cacng2* constructs (mean ± S.E., *n* = 8/group) showing higher expression of the GA-58 and GA-60 constructs relative to the GA-50 construct

For the initial characterization of the rat *Cacng2* promoter, genomic sequence of different lengths (see Fig. 1) was amplified from Sprague Dawley rat genomic rat DNA using primers flanked with KpnI and HindIII restriction enzyme sites (see Table S1). Amplified products were purified, ligated into pGEM-T as described above and sequence verified. *Cacng2* sequences were then ligated into KpnI-HindIII-cut pGL4.10 (Promega). For the subsequent analysis of GA-repeat length variation, similar procedures were used to obtain “F11-R10” constructs containing sequence-verified GAERS and NEC rat GA repeats (respectively, 60 bp and 58 bp repeats). A previously characterized dominant-negative REST (DNR) expression construct (Park et al. 2007) was obtained by amplifying N-terminal mouse REST sequence with restriction enzyme-flanked PCR primers (Table S1; DNRESTF1 and DNRESTR2) and cloning the sequence-verified fragment into the expression vector pcDNA3.1.

For comparison with the *Cacng2* promoter constructs, we selected the synapsin I (*Syn1*) promoter, widely used as an experimental promoter with relative neuronal specificity (see Matsuzaki et al. 2014). In order to enhance comparability between the two promoter sequences with respect to species and sequence length, we used similar cloning procedures (see above) to obtain a de novo rat *Syn1* promoter construct (Supplemental data, S4; Genbank Accession: MH119182)*,* but included additional 5′ flanking sequence compared with the commonly used “core” promoter sequence (Matsuzaki et al. 2014; cloning oligonucleotides in Table S1).

Hippocampal HT22 cells (provided by JD) were maintained in DMEM (Invitrogen/Thermo Fisher) with 10% Fetal Bovine Serum (FBS, Invitrogen), and PC12 cells (Gift of Prof. D. Murphy, University of Bristol, UK) were grown in DMEM with 10% horse serum (Invitrogen) and 5% FBS. Both culture media were supplemented with 1x antibiotic/antimycotic (Invitrogen), and cells were grown on 12- or 24-well Costar CellBIND® plates (Corning, Kennebunk, ME, USA) at 37 °C, and in 5% CO_2_. For experimental analysis, cells (1 × 10^5^ or 5 × 10^4^ for 12-, or 24-well plates, respectively) were transfected (TransFast protocol, Promega) with plasmid constructs (PureYield, Promega protocol) and maintained for 24 h prior to either reporter assay, or, in some cases RNA extraction (see above). Plasmids were used at different amounts for 24- and 12-well plates (100 or 150 ng/well respectively), and were co-transfected with the control pRL-TK plasmid (5 ng, Promega). Where used, the DNR construct was transfected at 250 ng. In other experiments, HT22 cell cultures were treated with N^6^,2′-O-Dibutyryladenosine 3′,5′-cyclic monophosphate sodium salt (dbcAMP; 200 mM; Sigma) or vehicle for 24 h, and then RNA was extracted as described above. Transcription reporter assays were conducted with the Dual-luciferase reporter assay system (Promega), using a Luminometer (Model TD-20/20, Turner Biosystems, Sunnyvale, CA, USA). Each transfection was replicated either sixfold (3 replicates, in 2 transfection experiments) or eightfold (4 replicates, in 2 transfection experiments), as indicated in the “Results” section. Data was analyzed by first normalizing against individual pRL-TK values, and then calculating fold-difference, compared to the activity of “empty” pGL4.10 vector.

### Bioinformatics and Statistical Analysis

Rat *Cacng2* gene promoter sequence was predicted from flanking genomic sequence associated with mammalian *Cacng2* exons mapped on the UCSC genome browser (genome.ucsc.edu), and also with reference to predicted mouse and human promoters on the Eukaryotic promoter database (epd.vital-it.ch). Species and strain sequence variants were obtained from the STR catalog viewer (http://strcat.teamerlich.org; Willems et al. 2014) and The Mouse Genomes Project (ensembl.org/Mus_musculus/Info/Strains). Potential trans-regulatory sites were identified using the Meme suite (meme-suite.org/) and Lasagna (biogrid-lasagna.engr.uconn.edu/lasagna). Potential regulatory sites in the *Cacng2* 3′ UTR sequence were searched with “AREsite” that detects AU-rich sites (rna.tbi.univie.ac.at/cgi-bin/AREsite.cgi; Gruber et al. 2011), and TargetScan for conserved miRNA target sites (www.targetscan.org). Sequence identity was initially confirmed using BLAST (blast.ncbi.nlm.nih.gov), and sequence comparisons were conducted with Clustal Omega (ebi.ac.uk/Tools/ msa/clustalo). Statistical analysis was conducted with IBM SPSS Statistics version 20 (IBM, New York, USA) by applying different tests as indicated in the Results, and accepting *p* < 0.05 as the significance level.

#### Data Availability

The Genotype-Tissue Expression Project (GTEx) data used for the analyses described in this manuscript were obtained from the UCSC Genome Browser (http://genome-euro.ucsc.edu) on 02/10/2017.

## Results

### Structure of the *Cacng2* Promoter

Putative rat *Cacng2* promoter sequence was identified by selecting genomic DNA sequence that flanks the start of rat CACNG2 coding sequence, and also aligns with human and mouse promoters that are predicted on the Eukaryotic Promoter Database (Supplemental data, S1). A number of prominent features were identified in the selected 1832 bp of sequence including an initiator sequence, multiple RE-1 silencing transcription factor (REST/NRSF) elements, and a short tandem repeat (STR) sequence, that consists of a run of repeated GA motifs (Fig.1; Supplemental data, S1 & Fig. S2). With reference to the conserved CaRE element previously identified in mouse sequence (Pfenning et al. 2010), it was also possible to identify an equivalent rat CaRE sequence (Supplemental data, S1).

The genomic DNA sequence identified above (Supplemental data, S1) was cloned and sequenced from GAERS, NEC (Non-epileptic control strain) and Sprague-Dawley rats. No inter-strain sequence variants were observed with the exception of variation in the length of the STR sequence (Table 1, Supplemental data, S2). The latter finding is, in fact, consistent with recorded sequence variation already lodged in the nucleotide and genome databases. In fact, sourcing these sequences from different databases reveals that multiple length variants are recorded at both inter- and intra-species levels (Table 1). Our sequencing results also indicate some intra-individual variation; for example, values of either 50 nt or 52 nt for the Sprague-Dawley strain (Table 1). Both these values accord with the value of 50 nt for Sprague-Dawley sequence in the NCBI nucleotide database (e.g., XR_601918.1), and the small differences may relate to either some degree of intra-individual variation/heterozygosity, or possibly, PCR “slippage” in the analysis (Clarke et al. 2001). Overall, however, we have observed a consistent strain variation, with a marked difference of up to 12 nt between the GAERS/NEC strains and SD rats. For an “in-house” species comparison, we also sequenced the GA-repeat region from CD-1 mice, finding values that approximated to many of the recorded strains in the Mouse Genomes Project database (Table 1).

**Table 1:** *Cacng2* gene variants at the poly-GA STR sequence

Species and strain	STR Length (current study)	Genome/database	STR length (genome/database)
Rat BN	–	Rat genome, rn6 XR_593643	54
			54
SD	50, 52, 50	XR_601918.1	50
GAERS	60, 60, 62		–
NEC	58, 58, 58		–
Mouse C57BL/6J	–	Mouse genome, mm10 XR_875456.1	48
			48
CD-1	38, 38, 40		–
sPRET/EiJ	–	MGP	30
pWK/phJ	–	MGP	38
cAST/EiJ	–	MGP	30
wSB/EiJ	–	MGP	40
nZO/HILtJ	–	MGP	34
C57BL/6NJ	–	MGP	48
nOD/ShiLtJ	–	MGP	42
fVB/NJ	–	MGP	40
dBA/2 J	–	MGP	42
cBA/J	–	MGP	40
c3H/HeJ	–	MGP	40
aKR/J	–	MGP	42
bALB/cJ	–	MGP	40
a/J	–	MGP	40
IP/J	–	MGP	40
129S1/SvImJ	–	MGP	42
Human	–	Human genome, hg38	66
	–	STR catalog viewer*	51, 53, 55, 57

Values are GA-repeat length in nucleotides (e.g., 50 = 25 × GA). Values shown for the current study are representative of individual biological replicates

*Willems et al. (2014). *BN*, Brown Norway; *GAERS*, Genetic Absence Epilepsy Rat, Strasbourg; *NEC*, normal epileptic control; *SD*, Sprague-Dawley. Mouse strain nomenclature is from the Mouse Genomes Project (MGP, see “Methods” section)

In an additional sequencing analysis, we also showed that 3′ UTR sequence in GAERS *Cacng2* mRNA did not exhibit sequence variation when compared to the genome database (Supplemental data, S3). Taken together, these sequencing results provide no evidence for sequence variation within the proximal regulatory sequence of GAERS rat *Cacng2*, with the notable exception of a length variation at a poly-GA STR sequence.

### Functional Domains of the *Cacng2* Promoter

In further experiments, the *Cacng2* sequence identified above was cloned into a promoter-less vector (pGL4.10), and directly analyzed for promoter activity using a transient transfection approach in two cell lines, HT22 and PC12. For comparison with another neuronal promoter sequence that has been extensively characterized for other species, the rat *Syn1* promoter was similarly cloned (Supplemental data, S4) and tested in parallel. Initial studies showed that the rat *Syn1* sequence strongly drives transcription of the luciferase reporter gene in HT22 cells, resulting in approximately 50-fold induction compared with empty vector (Fig.1; *p* < 0.05, Student’s *t* test for independent samples, *n* = 6/group). Subsequent experiments utilized a number of different sized *Cacng2* promoter constructs, and all of these constructs were found to be markedly less active than the *Syn1* construct, driving only modest levels of transcription even following the selective removal of multiple different sequence blocks (Fig.1, see details below). Statistical analysis showed that only four *Cacng2* constructs (named 1234 bp, 801 bp, 778 bp, and 661 bp; Fig.1) stimulated transcription above the control (vector) level (*p* < 0.05, Student’s *t* test for independent samples, *n* = 6/group; Fig. 1). These findings were also broadly confirmed (using selected constructs) in PC12 cells where the *Syn1* promoter was again markedly more active than *Cacng2* constructs (fold-difference vs. empty vector: SynI, 50.8 ± 4.6; Cacng2 801 bp, 9.6 ± 1.0; Cacng2 1234 bp, 3.6 ± 0.5; *n* = 6/group).

The extensive promoter analysis conducted in HT22 cells revealed three repressive regions: a distal, upstream region containing the identified CaRE element, a larger distal region containing the multiple REST elements, and a third, downstream repressive region (Fig. 1). Evidence for independent regulatory activity in these three regions can be found in the statistical comparisons between: 1234 and 1344 bp (deleting CaRE region), 801 and 1234 bp (deleting 3 REST elements), and 780 and 661 bp (deleting downstream region). For each of these comparisons, ANOVA and post-hoc analysis conducted across all *Cacng2* constructs (Fig. 1) revealed significant differences in fold induction (*p* < 0.05, one-way ANOVA, and Dunnett T3 test, df (6.35), *F* = 37.789, *n* = 6/group). Our inference of independently acting repressive elements is also generally supported by additional comparisons between different constructs in this analysis (Fig. 1). The absence of any significant difference between the 661 and 801 bp data indicates that the remaining REST consensus sequence in 801 bp does not significantly influence activity, at least in this experimental context. In order to directly evaluate the role of the full array of REST consensus sequences in this promoter context, a dominant-negative REST construct (DNR, see “Methods” section) was co-transfected with the *Cacng2* 1234 bp construct in HT22 cells (Fig. 1). Marked over-expression of this DNR sequence was demonstrated by RT-PCR analysis of transfected cells (Supplemental Fig. S5), and this was associated with a significant increase in transcription from the *Cacng2* construct, indicating derepression of the promoter (Fig. 1). In this experiment, ANOVA and post-hoc analysis between the different experimental conditions revealed a significant difference in fold-induction when the DNR construct was co-expressed with the 1234 bp *Cacng2* construct (*p* < 0.05, one-way ANOVA, and Dunnett’s T3 test, df (3.20), *F* = 34.707, *n* = 6/group).

The *Cacng2* F11R10 1234 bp construct was also selected for analysis of the functional properties of the variable GA-repeat sequence. For this purpose, two additional F11R10 constructs were synthesized containing the longer 58 bp (NEC-representative) and 60 bp (GEARS-representative) GA-repeat length sequences. A three-way comparison between the GA-50 (Sprague-Dawley), GA-58, and GA-60 constructs in further transfection experiments revealed a modest but significant increase in activity of the GA-58 and GA-60 constructs compared with GA-50 construct, but no apparent difference in activity between the latter two constructs (Fig.1; *p* < 0.05 GA-50 vs both GA-58 and GA-60, one-way ANOVA and Dunnett T3 test, df (3.28), *F* = 31.592, *n* = 8/group). The GA-repeat sequence is therefore identified as having regulatory function in this promoter context. Additional analysis of this repeat sequence on the UCSC genome browser revealed a high level of conservation (Supplemental Fig. S6), extending far beyond that already identified for human, mouse, and rat genomes (Table 1 data).

### Co-regulation of *Cacng2*-Paired lncRNAs

**Fig. 2:**
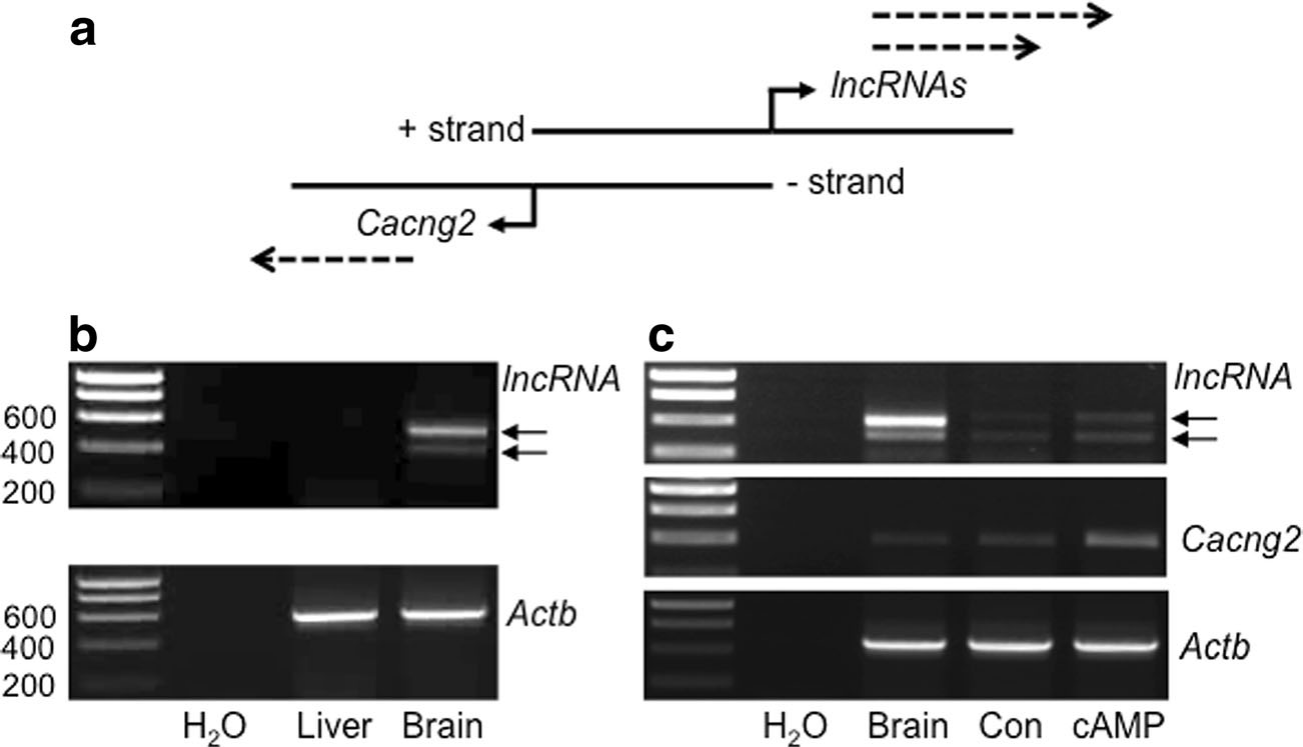
Tissue-specific and regulated expression of (antisense) lncRNAs associated with the *Cacng2* promoter. **a** Schematic representation (not scaled) of the *Cacng2* locus illustrating the “head-to-head” organization of *Cacng2*-coding and lncRNA(s) sequences in the rat genome. The dashed arrows indicate the generation of the respective transcripts from opposite strands of DNA, with multiple arrows indicating the presence of multiple lncRNA transcripts of different sizes, as indicated by the current data. **b**, **c** Representative images of agarose gel electrophoresis analysis of PCR-amplified *lncRNA*, *Cacng2* mRNA and *Actb* mRNA from different tissues and cells. **b** Tissue-specific expression of *Cacng2*-associated lncRNA in the brain. **c** Cacng2 and lncRNA amplicons are upregulated in cultured HT22 cells following treatment with dbcAMP (cAMP) as compared with vehicle-treated cultures (Con). Numbers on the left of gels are sizes in base pairs. Arrows indicate the two different lncRNA amplicons (see text). H_2_O represents a cDNA-negative control PCR reaction

Observation of the *Cacng2* genomic locus on the human and mouse genome browsers indicates a potential bidirectional promoter organization (Fig. 2a; Adachi and Lieber 2002), given the proximal, downstream location of lncRNA transcripts that are oriented “head-to-head” with *Cacng2* (human RP1-293 L6.1 and mouse AK043153; Supplemental Fig. S7A). Notably, GTEx analysis indicates an almost identical expression profile for human *Cacng2* and the associated RP1-293 L6.1 transcript (Supplemental Fig. S8). Analysis of human ESTs also reveals similar (brain-specific) expression of EST sequences in this genomic region (e.g., DA799098; Fig. S7B). This region also features an annotated CpG island in the mouse *Cacng2* promoter region that is highly conserved in rat (Supplemental data, S9), and CpG islands are common features of bidirectional promoters (Uesaka et al. 2014). In order to investigate the presence of expressed downstream sequences in rat, that are antisense to *Cacng2*, we designed primers (Table S1) to rat sequence equivalent to that of mouse AK043153, following BLAT analysis of the rat genome, and used these primers to amplify cDNAs from oligo-dT-primed rat RNAs (Fig. 2). Abundant PCR products were derived from rat brain but not the liver (Fig. 2B), a consistent result that was replicated from independent tissue samples (data not shown). The two brain-derived product bands were cloned and sequenced, revealing multi-exon RNA sequences (405 bp and 578 bp; Supplemental data, S10) with relative homology to both human RP1-293L6.1 and mouse AK043153. However, the cloned rat RNAs have an exon structure that is distinct from current database sequences, because whereas the two terminal exons map to annotated mouse exons in AK043153, for example, the intervening exons, although highly conserved at a sequence level, do not. Clearly, this distinction may relate to currently incomplete transcriptome annotation rather than actual species differences in transcript structure. The two novel rat RNAs cloned in this initial analysis (405 bp and 578 bp; Supplemental data, S10) have accession numbers: MH340060 and MH340061.

Following the demonstration of brain-specific expression of *Cacng2*-associated rat lncRNAs, the regulation of their expression was investigated in (mouse) HT22 cells, and compared with that of *Cacng2* mRNA (Fig.2c). This analysis involved a cAMP “differentiation” paradigm (Inda et al. 2017) in which HT22 cells were treated with the cAMP analogue, dbcAMP. Initial end-point RT-PCR analysis revealed that PCR primers designed against the equivalent mouse sequences used for rat lncRNA permitted amplification of similar products, as would be predicted from sequence conservation (see above; Fig. 2c). However, these products can be clearly observed only in the dbcAMP-treated samples, indicating, firstly, a low level of lncRNA expression under basal conditions in these cells, and secondly, regulated expression, in this case by cAMP. Similar results were observed for HT22 *Cacng2* mRNA, indicating that these transcripts can be co-regulated. Subsequent QPCR analysis confirmed co-regulation quantitatively, with both lncRNA and *Cacng2* amplicons showing significant fold-increases in dbcAMP-treated cultures compared with controls (2.61 ± 0.45-fold and 2.57 ± 0.29-fold, respectively, *n* = 6, *p* < 0.05, Independent samples *t* test for both amplicons). Interestingly, however, the control lanes used for the end-point PCR analysis (Fig. 2c) revealed an apparent higher level of lncRNA transcripts relative to *Cacng2* transcripts in whole brain, suggesting that although co-regulation can be observed, there is also an indication of independent lncRNA expression (in selective, unknown brain areas), an observation that requires investigation in further studies (also, see below).

### Bidirectional Activity of the *Cacng2* Promoter

**Fig. 3:**
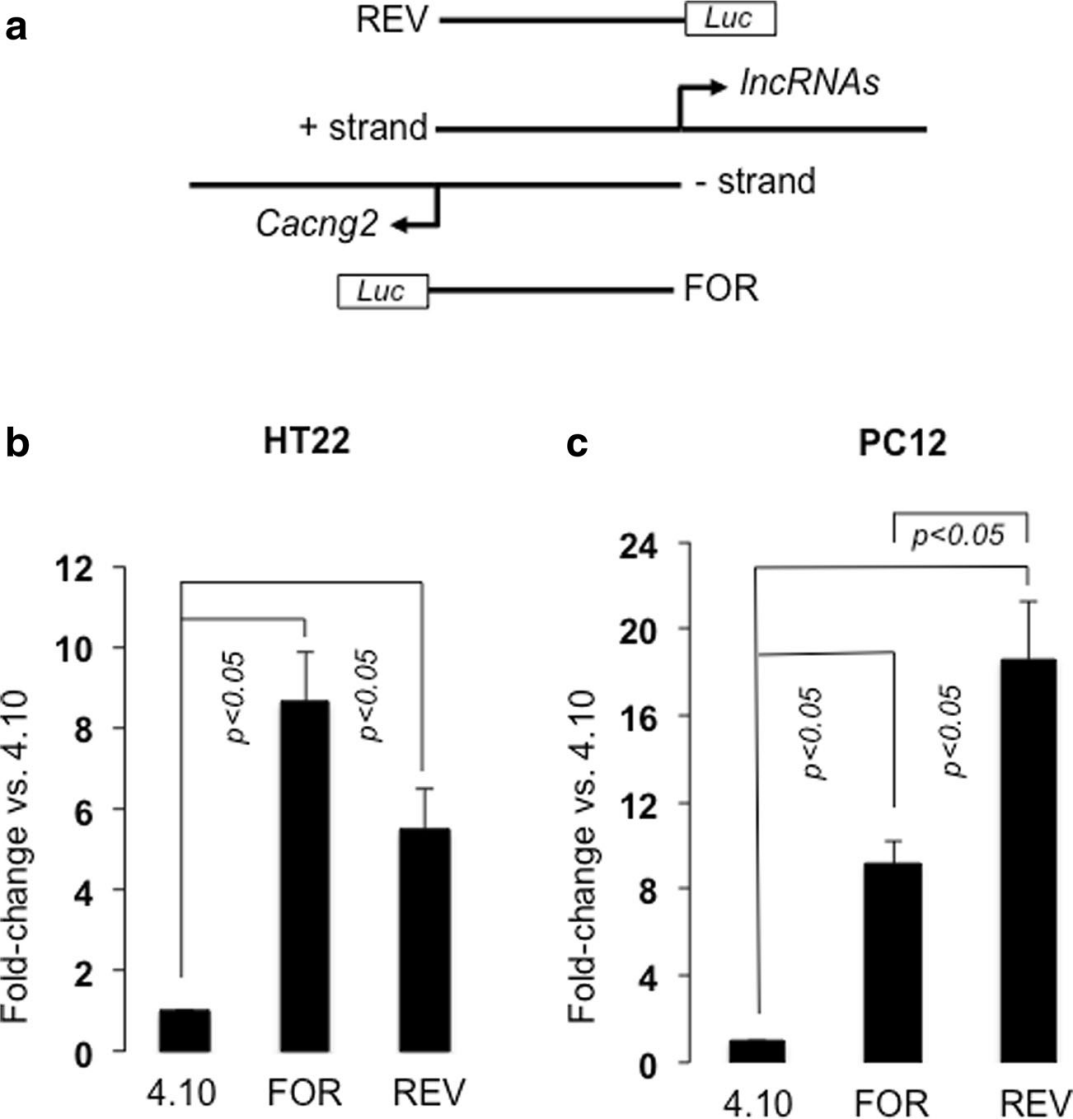
Bidirectional activity of the *Cacng2* promoter activity in transfected HT22 and PC12 cells. **a** Schematic representation (not scaled) of the *Cacng2* locus illustrating the “head-to-head” organization of *Cacng2*-coding, and lncRNA(s) sequences in the rat genome, and the forward- (FOR) and reverse-orientation (REV) constructs used in the experiments. **b**, **c***Cacng2* sequences were cloned within pGL4.10, transfected into either **b** HT22 or **c** PC12 cells, and levels of expression (fold-change relative to empty pGL4.10) were determined by luciferase (Luc) assays. *p* < 0.05 indicates statistically significant difference between different groups as determined by ANOVA and post hoc analysis (see text). The data are expression levels of constructs (mean ± S.E., *n* = 6/group)

In order to further investigate the potential bidirectional organization of the *Cacng2* promoter with associated lncRNA transcripts, further RT-PCR and transfection studies were performed. Initially, RT-PCRs were designed to identify more 5′ lncRNA sequence. This analysis was based on the genomic location of a (+ strand) rat EST (AI535545.1), and a similar mouse AK043153 RNA that are both initiated upstream of the rat lncRNA transcripts identified above, therefore indicating proximity to the *Cacng2* promoter. Using primers RlncF2 and RlncR3 (Table S1), a PCR product was again consistently derived from rat brain but not liver (Fig. S11). Cloning and sequencing of the PCR product revealed a 491 bp sequence (Supplemental data, S10) that mapped to the predicted region of the rat *Cacng2* locus, and was contiguous with the initial exon sequence of the two rat lncRNAs identified above. This result indicates a long initial lncRNA exon in rat that is similar to that seen in the mouse AK043153 RNA, and is also partially overlapping annotated ESTs (mouse: BB640458 and BB643739; rat: AI535545, BF522394, and CB708921). Therefore, the lncRNA(s) are initiated (at most) just 594 bp from the *Cacng2* initiator sequence, and, given the location of AI535545, possibly as little as 555 bp. Taken together with our *Cacng2* promoter analysis, this sequencing confirms the predictions of current genome annotation (see above), namely that the (reverse-oriented) *Cacng2* and lncRNA transcripts are initiated proximally, and may therefore share a bidirectional promoter. To test this hypothesis, further transfection experiments were conducted using an additional (reverse orientation) construct based on the 801 bp *Cacng2* promoter construct that includes the initial 25 bp of the 5′ lncRNA sequence identified above. Using both HT22 and PC12 cells, it was shown that the 801 bp promoter sequence significantly enhanced transcription in both orientations, providing strong, functional evidence for the presence of a bidirectional promoter in this region (Fig. 3; HT22: *F* (2.17) = 16.781, *p* < 0.05, Dunnett T3 test; PC12: *F* (2.17) = 30.298, *p* < 0.05, Dunnett T3 test; *n* = 6/group). Interestingly, the reverse orientation construct was relatively more active in PC12 cells, driving significantly more transcription than the (forward (*Cacng2)*-oriented) construct in this cellular context (Fig.3c; *p* < 0.05, Dunnett T3 test). Further studies are required to identify the possible physiological significance of this finding, but clearly, the promoter can be relatively more active in this orientation, which is interesting given the high levels of brain lncRNA (relative to *Cacng2*) transcripts identified in our previous RT-PCR analysis (Fig.2c).

## Discussion

Differences in gene expression level between conspecifics is a recognized determinant of mutant phenotype severity (Vu et al. 2015), and one source of this variation is STR (microsatellite) polymorphism (Hannan 2018; Willems et al. 2014). In humans, polymorphic STR loci (Willems et al. 2014) correlate with many different phenotypes including human behaviors that are associated with psychiatric disorders (Bagshaw et al. 2017; Landefeld et al. 2018). In the current study, we have made the novel observation that a GA-repeat STR within the *Cacng2* promoter region is a candidate sequence for this type of function-related polymorphism. Here, we show that this GA-repeat is highly variable both between and within species. In different rat strains, we detected multiple different variants in this neuronal promoter region, a finding that reflects previous analyses of promoter-associated repeats in rodents (Kacew and Festing 1996). In addition, we have shown that natural polymorphisms affect activity of the *Cacng2* promoter that has been defined here for the first time.

An absence epilepsy model-related GA-repeat variation (NEC vs. GAERS rats) was also identified, but was not found to correlate with *Cacng2* promoter activity in the current experiments. This negative result may simply reflect the in vitro/cell culture context of these studies. At the same time, it is also the case that the GA-repeat variation is but one (potential) contributory genetic factor in this polygenic animal model (see Cope et al. 2009; Holter et al. 2005a; Powell et al. 2009), and therefore a GA-repeat-related contribution may be subtle, but nevertheless of significance when manifested in combination with other variations in different contributory genes. Previous studies have shown that small variations in repeat length alleles can contribute to phenotype within an appropriate context; for example, a 12 vs 13 AC-repeat variation in the *Tbr1* gene promoter has been shown to correlate with a human behavioral phenotype (Bagshaw et al. 2017). Notably, the *Cacng2* GA-repeat variation is extensive across different mouse strains and may contribute to other phenotypes, including other epilepsies. With respect to generalized epileptic seizures, this variation may be of relevance because there are marked differences in seizure susceptibility between different mouse strains (Kosobud and Crabbe 1990), including kainic acid-induced seizures (McKhann et al. 2003), and *Cacng2* is a determinant of kainic acid responsiveness (Tomita et al. 2007). However, inspection of the current list of *Cacng2* GA-repeat STR variations across mouse strains (Table 1) does not indicate a simple correlation between repeat length and kainic acid-sensitivity, because although C57 mice, for example (repeat length, 48), are markedly more tolerant of kainic acid than the 129/SvJ strain (repeat length, 42), C3H mice, on the other hand (repeat length, 40), have a kainic acid-sensitivity level more similar to C57 mice than to the 129/SvJ strain (McKhann et al. 2003). Further functional analysis of *Cacng2* repeat length in different mouse strains is required, noting that other genetic differences between these strains must always be considered when making these individual genotype-phenotype correlations.

With respect to promoter dinucleotide polymorphisms in general, these are recognized to be correlated with gene expression (Bilgin Sonay et al. 2015), being observed in numerous promoters (Sugiyama et al. 2011; Ohadi et al. 2012; Nikkhah et al. 2015; Liu et al. 2015; Emamalizadeh et al. 2017), and forming potential “tuning knobs” of gene expression (Abe and Gemmell 2016; Sawaya et al. 2012, 2013; Vinces et al. 2009). The reported effects of STR variation are gene-specific, resulting in either enhanced (e.g., Sugiyama et al. 2011) or reduced (e.g., Liu et al. 2015; Chen et al. 2016) promoter activity, as a function of increasing dinucleotide repeat length. These different outcomes are clearly related to promoter-specific structure-function relationships that could involve many factors including promoter topology (Philips et al. 2015), spacing of transcription factor (TF) sites (Bagshaw et al. 2017), or methylation differences where there are CpGs (e.g., C9orf72; Gijselinck et al. 2016; see below). The mechanism involved in mediating the observed differences in activity between *Cacng2* GA-50 and *Cacng2* GA-58/60 is unknown, but may involve TF binding. In *Drosophila* sp*.* two zinc finger proteins, GAF (GAGA factor) and CLAMP (Chromatin-linked adaptor for MSL proteins) bind GA repeat sequences and regulate gene expression, for example, at a bidirectional histone promoter (Rieder et al. 2017). In the latter example, there is a defined functional correlate of repeat length where TF binding is enriched at long GA repeats on the X chromosome, mediating dosage compensation in males (Kuzu et al. 2016; Rieder et al. 2017). A functional equivalent of GAGA factor in mammals is the ETS family transcription factor GABP1 (GA binding protein-1; Yang et al. 2007), and further experiments are required to determine whether this factor could be active at the *Cacng2* promoter. Alternatively, other studies (Chen et al. 2016) indicate a need to consider STR length-related differences in TF binding outside of the repeat sequence.

The current study has also provided experimental evidence of a role for the zinc finger transcription factor REST (Chong et al. 1995) in regulating *Cacng2* expression through a promoter-proximal mechanism. Both deletion of a proximal, multi-REST element domain and over-expression of a dominant-negative REST protein significantly elevate expression of rat *Cacng2* constructs. These experimental outcomes are quite extensively supported in the literature, with three genome-wide studies identifying *Cacng2* as one of the > 1000 REST target genes in the genome (Bruce et al. 2004; Johnson et al. 2007; Otto et al. 2007), and additional transcriptomic studies in PC12 cells indicating direct regulation of *Cacng2* by REST (Dijkmans et al. 2009; Garcia-Manteiga et al. 2015a, 2015b). Further studies are required to confirm the function of the individual *Cacng2* REST consensus elements identified in our sequence analysis, and also to investigate a potentially interesting association of these REST elements with epileptic phenotypes (Thiel et al. 2015). Analysis of ENCODE ChIP-Seq data (www.encodeproject.org*)* shows that conserved *Cacng2* REST sites in other species are associated with additional repressive associations, including Sin3A and HDAC, providing a basis for powerful, co-repressor-dependent silencing of this neuronal gene (Lunyak et al. 2002). In marked contrast to the presence of multiple consensus REST target sequences in the *Cacng2* promoter, the rat *Syn1* promoter cloned and employed here does not contain searchable REST sequences; this absence probably partly explains the relative activity of the *Syn1* promoter compared with *Cacng2* in these experiments. Previously, identified consensus REST sequences in the human and porcine *Syn1* promoter (Hedegaard et al. 2013) are not sufficiently conserved in the rat sequence, and residual sequence may exhibit only weak REST binding (Bruce et al. 2009). It is likely that the rat *Syn1* gene utilizes alternative REST sequences outside of the proximal promoter region, for example, in an intron like the human synaptophysin gene REST element (Lietz et al. 2003). An alternative *cis*-regulatory distribution of sites would accord with the general observation that functionally conserved regulatory elements are not necessarily positionally conserved across genomes (Cheng et al. 2014).

We have also demonstrated that the rat *Cacng2* promoter has a bidirectional organization (Adachi and Lieber 2002), involving antisense transcription of associated lncRNA(s). Bidirectional activity of the identified *Cacng2* promoter sequence was functionally demonstrated here, using forward and reverse promoter constructs as for other, similarly oriented, gene pairs (Jiménez-Badillo et al. 2017). Our finding is consistent with both the observed proximal initiation of the paired *Cacng2* and *lncRNA* transcripts (generally within 2 kb in bidirectional promoter-associated, neuronal gene pairs, Hu et al. 2014), and also with features such as the central CpG island (Uesaka et al. 2014), and the absence of a TATA box (Lasagna Transfac analysis; Bagchi and Iyer 2016). Current genome annotation indicates that bidirectional *Cacng2/lncRNA* transcription is conserved across rat, mouse, and human, but our current sequencing data from rat indicate that there may be some degree of species-specific exon usage in the lncRNA(s), a common observation for these non-coding RNAs (Clark and Blackshaw 2014). In fact, bidirectional promoter organization is now recognized as a norm for neuronal genes (Hu et al. 2014) and is a major source of lncRNAs (Uesaka et al. 2014; Hon et al. 2017). Further studies, which may be extensive, are required to delineate the full exon structures of the different transcripts that emanate from *Cacng2*-associated lncRNA start site (see Fig. S7B for current human annotation). Here, we have provided in vitro evidence for co-regulation of *Cacng2* and the associated lncRNA, data that is consistent with both single cell transcriptomic analysis in developing human cortex (Liu et al. 2016), and the near identical expression profiles of these transcripts in the human GTEx platform (Supplemental Fig. S7A). Together, this data provides evidence of regulated co-expression of the mRNA:lncRNA pair which has been observed in other, similarly organized, genes, and can form an aspect of transcriptional regulation (Kaur et al. 2016; Yamamoto et al. 2016; Jiménez-Badillo et al. 2017; Malhotra et al. 2017). Currently, we have no evidence of a regulatory inter-relationship at this bidirectional promoter site. The demonstrated CpG island in the *Cacng2/*lncRNA promoter region is one regulatory sequence that should be investigated in further studies as these features are commonly found in head-to-head bidirectional promoters, and are known to be subject to demethylation by the linked lncRNAs (see Uesaka et al. 2014). In considering the functional activity of these lncRNAs, however, alternative *cis*, or *trans* activities at other genes must also be considered (Clark and Blackshaw 2014).

Overall, the current analysis has shown that multiple regulatory sequence domains, including those likely to determine neuronal-specificity, and which are commonly distal (i.e., REST; Bruce et al. 2004) reside proximal to the *Cacng2* start site. This organization may indicate merging of regulatory units into a dual promoter-enhancer, “dyadic’”organization that is now recognized (Roadmap Epigenomics Consortium 2015). Given the established role of CACNG2 in neural plasticity, the presence of a polymorphic, regulatory STR in this promoter region could be important for determining differential plasticity between individuals (Lee et al. 2016; Louros et al. 2014). In addition to the aspects of pathology discussed above, this could also include a contribution to differences in pain-related plasticity in which CACNG2 is implicated (Nissenbaum et al. 2010; Sullivan et al. 2017). Hence, in common with other (regulatory) polymorphic repeat sequences (e.g., Kotur et al. 2015), the *Cacng2* STR is clearly a potential marker that could be widely applicable to pharmaco-genomic analysis (Daly 2017). Our characterization of this synaptic protein gene promoter may also be relevant to the refinement of neuronal promoters that are required for current transgenic targeting strategies (see Holehonnur et al. 2015).

## Electronic supplementary material

ESM 1 (DOCX 737 kb)

ESM 2 (XLSX 34 kb)
