# Supplementary material for: Regulatory Architecture of the Neuronal Cacng2/Tarpγ2 Gene Promoter: Multiple Repressive Domains, a Polymorphic Regulatory Short Tandem Repeat, and Bidirectional Organization with Co-regulated lncRNAs
Source: J Mol Neurosci. 2018 Nov 26;67(2):282–94. doi: 10.1007/s12031-018-1208-x (PMC6373327; doi:10.1007/s12031-018-1208-x)
Supplement: Supplementary file 1 — (DOCX 737 kb) [file 12031_2018_1208_MOESM1_ESM.docx]

**Regulatory architecture of the neuronal *Cacng2/Tarpγ2* gene promoter: multiple repressive domains, a polymorphic regulatory short tandem repeat, and bidirectional organization with co-regulated lncRNAs.**

**B.P.A.Corney, C.L.Widnall, D.J.Rees, J.S.Davies, V. Crunelli, D.A. Carter.**

**Supplemental Information.**

**S1. Cloned rat genomic DNA sequence showing the location of some specific conserved regulatory elements, and the (variable) dinucleotide repeat (STR) sequence.** This genomic sequence is derived from the GAERS rat genome and includes a 60nt GA repeat that is distinct from other strains (see Table 1). The lower sequence block, which is flanked by PCR primers CacnF6 and CacnR10 (see Table S1), spans the entire sequence used in promoter analysis (see Results, and also note differences in GA-repeat length in different strains used). The upper block of sequence shows additional upstream sequence cloned from GAERS DNA (starting from CacnF1) for sequence verification, but not used in promoter analysis.

Rn6: chr7:119,352,052-119,353,877

GGAAACTTGGAAAGATCCACCTACTTCTCCAGAAAACATTTTTATCTTTGAGATGAGTATTATAGCCCAAACCCCAGTCACCAGCCACCCTCCTTCTCATTCCTTCACAAGAATAATCTTTCCAAAATGTCTACTTGTGGAAGCAAGATGTAGAATTTCACAGAAAAGCTACAGAGCCACAGATAAAGCTATGCACACCTCTGTTAGCCAGAAAGAAAGATCAAGAAAAAAATGAGGAAGGAGATGCATCTATCCAGCCAGTAAAATAAAACAACCTCCCCTCCCCAAAAAGTGTGGAACCCATGCTTAGGGGAGGAAGAAAAGCACATAGAGACTATGTTTGTAATTACCCATCCAGG

TTCTCTTCCAGGAGGAGAAGCAGCTGATTGAAGTTTTGTGAGAGTCTGACTGTGATGGGAGGGCTGTATTTGAAGCTCTGGGCACGTAGAGTTGGAATGAAAAGGAGGCTGATCTGGAAGAAACACCAAGGGGGGAGGGAAGTGGAGAGAGAGAGAGAGAGAGAGAGAGAGAGAGAGAGAGAGAGAGAGAGAGAGAGAGAGAGATCGCCAGCTACGCCTTCCTCCCAGCTCCAGGAGGCCCTGGACAGCGCTCATTCCGGAACTGTGGAACAGGAGTCGGGGAGGCCAGGCAGCCCGGCCTGGCCCGTCCGCGGACAACAGAGGCCCCGTAGCCCGGCCCCACTCCTGGCGGGCTACAGAGCGCTGGGAGCGCACAGAGTGGGGGAGCTGTCCAGTTCAGCACCACGGCGACGCAGTAGGTGGGCGCTCCTGCTCCGAGAGCTGTCCAGTCCAGCACCGCGGCGATGCTGTAGGTGAGAGCGCCCTGTCTCGGGGAGCTGTCCAGTTCAGCACCACGGCGATGCAGTAAGTGGAAGAGCCAGGCCGGGGTGAGCTGTCCGTTTCAGCACCACGGCGATGCTGTAGCTGGGAGCGCCTGGCCCGGGGCTGCGCGGCCGCGAGCGGGGGAGGCGGGCGGGCCTGCCTGCTGCAAGACGCCGGCTAGCGCGCCCCATCGCTCTCTCGCTCGCTGGCTTCCAAGGAGCTTTGCGGATCGGGGGAGGGAACCCCGCCTCGATTCTCCGGGAGAAACCCCCTCCCTCCCCCACTTCGCCGACACTACCCCCGCCGCCAACAACAATAACCGCGGCTGCTCGTCCTGCCGCCACCGCGCCCGCCAACCCTGCTGCCGCCGCCGCCGCCGCCGCCGCTGCCGCCGCCGCCGCCGGGGCACAACCATGGGAGGCGAGACCCCCTCACCGGTGCAGCTGAGCTCGAGGGCTTGCAGAAGACTGGGCAGTCTCCTCTGAAACGGAGGCACCCCGAGGACCCCCTCCCTCCCCAAGGAAGAGAACTGGCGAAGCCACGGATTTTCATGGCGCAGGCCGCAGAGCCGCGAACGAAGACCGAAGGTTGCTTCCCAGCTCTTCCAGTCTCTAACTGTCTAGGTCCGGACCATGAGAGATGGTGTTGAGAATTCGGCTGTACCCTGGCGCGCTGTGGAAGCCATCTCCAAATTAGCGATCACATATGGAAACTGGAGACCAGAATTTTAGGAAAAGAGATTAAGGCATCTCACTTGGGGGGGGTGGGGGGTGTCTTTTTATTTATTTTCCCTTTTTTAAAAAAAATCGCTGCAACTGGAACAGTTTTTTGATCTCAAAAGGCAAGCCTCTCTTCCCGTGTGATCTTTATAATTTACACACTTTTCCGTGAGCTTTCTTATCTCCCTTTTTTTATATCTCTCCATATTCTCTATTCACACATATATCCATTATATTAGTAGTGGAATTACCATTCGCACCCTCACACACA

AAAAGGAGGC CaRE element

GAGAGAGAGA GA dinucleotide repeat

TTCAGCACCA REST/NRSF elements (x4)

CCAAATT Initiator element

GGAAACTTGGAAAGATCC CacnF1 primer target sequence

CCATTCGCACCCTCACACACA CacnR11 primer target sequence

**Fig. S2.**

**
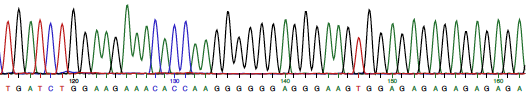
**

**
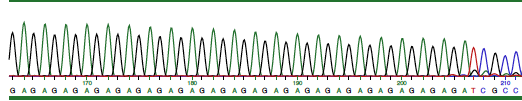
**

**Fig. S2. Partial electropherogram showing one example of length variation at the GA dinucleotide repeat sequence.** The sequence shown is from a NEC rat, and exhibits 29 GA repeats, ie. 58nt. In this selected example, the sequence exhibits relatively minimal ‘ski-slope’ effect in which the signal strength can diminish markedly across the repetitive DNA sequence.

**S3. Rat cDNA sequence of *Cacng2* 3’ UTR derived from RNA sampled from GAERS rat brain.** Rat 3’UTR sequence not currently annotated for the rat transcript (Cacng2-201 ENSRNOT00000008414; lower case text) was predicted by homology with the more extensively annotated mouse transcript (Cacng2-001, ENSMUST00000019290). In the current study, sequencing was restricted to the proximal 1573 nucleotides of the predicted 3’ UTR (flanked by primers, yellow highlight, Table S1), as this region was most highly conserved, and contained multiple predicted ARE elements (green highlight), and two conserved miR-128 target sites (blue highlight).

ACCACGCCCGTATGAAGACCGTGGGACGGGGAACCCCCGGGAGGCTTGGCCCGCGGGC

GGGGGAGGGACCACAGCAGCCACGGGGAGACCTTCCATACGCAAAAACAAAAAACAAA

AAAAAAAAACAAAAAAA

aaacaaaacaaaaaacaaacaacaaacaagcaaacgaacgaacaaccaacaaacaaaaca

aaaaacaacaaaaaaagagaaaaacatagtaagtaaatttaaaaaaaaaaaagaacaaac

tacaagaagaacaaaatcaaaaccactggctatgcggggaaaatataaccgagggaagac

aaactttaaacaaagcaagagggataaaattaaaatagaaaataaatctaaaagaaaatg

catgatttcccatgtaccattattttaacatttaataaaaaccaatttaaatgaaaaaaa

ataaaagggaaccaagataatgtaaagcaaaaaaacaaaaaacaacaaaaaaaaacttta

aaaaaacaaaaaaaatgaggaaagcaagagagtatttattagggttttatgttgcctttt

ttgtttctgttttttgttttaactgcagggagagtttaaaaataaataaataaataaatt

taaaacgcgttcttttccctttaaccccagcgggccctgcctcccggggagagtaggggg

tagagactcagggcccccagggccaggggagcaatggtcactgccaagcccctggatctc

tggctggaccctgggatgctggagaagttcaatggctttgcggccaactctatccagcac

taatggggcagttgcaggcctccaggtgacccaagcccttggtccgcccctatccgttag

gtgcctggaggtggtgcacttggggcttccccggggccccaggttcccagtccttaatgg

tccttaacccactgtgatgacttcctaggccttgtggaaagggaaggagaaggaatggct

gctagtggctcacagagaagccagacccctggaatcctcagggggagccttcggtggggc

cagtaaccccgaggtcctgtccttggaggcaggagacacccacccccaggggatatagag

cagccctccctcctcacccctcacctcagggccactagatgaccctggggtgatggtgga

cccccacattcacaggcccccggccccctgggaagggggctctttgaccctttgggggtc

cgtggactcactgatgcccccctgggggcccggggagtccaacaatgacattgcaaaaag

gtttctttttacaaaagaaaaaggaaaaaacaagtggtgatttttttttaaataaaaaaa

ccacagactataaataaatgtaaatatataataagtgaatttacttgcaagaaaatcaag

tagtatttttttcttttgattcttttccagctttaaactgtgaaaacaaaaatcggggtg

gggtgggggacttaaaatttagcagggaacttgtaaagacaaaacaaaacaaaacaaaac

aaatatagaaaccattaaaacaaacaaacaaaatccattgggagagatgtgagctgggca

cagagccagagggaaaggagaaaggcccctacatcttccttgggactggagaaagagtct

cttttgcctgcagggaggtgctgttgatggagagaggagacacacacacacacacacaca

cacacacacacacacacacacacacacactcttcttcctacagtcacacccacagccact

gccccctaggctcagtcctgcagtcacagcccaagttcaccgctctatgaaaaccattcc

cattcaacacacagtcaggagtgtggctcagtggcagctgacctgtgccccaaagccatg

gaaaccagcccaactctattgctggcctttgtgctcctgcgtgccccctacccccatacc

ttgcatatgtgtggatgcaccacattcttgtacctggaagggctccaatctgtagtctag

acgtcatggctgtgtggcctctctgggcctcagcttcccactgtgtcaccttgtggacac

acagacttgagctccaaagggctcattcttatcttttgtgtctgtgtgtgacttttaaga

tctgccaccagaccagctcagcggaaaatggccaggagcaatcaaagcagtccggatagc

attaagaacatttctaaatttctctctctctctctctctctctctctctctctctctctc

tctctctcacacacacacacacacacacacacacacacacacaccatgtgcctcctcact

gtgttccccagaccccaaccccacatccagtgtcaaagcaaaaattcacacacgagaaca

acctaccctgccatgcagacagagggagatgcgcagttccaaaagtcctcagcacgcctt

ccctttctgagtgagtgggggagggaggcgtctttgagagactatctcctgctatctttg

aggtcagtgaaaaagcaggccttcttggatggaagatctccctgtgtggaagaaatgttt

gcagaatactctccagagcaagtaccaaccgggtcaggcctgcgccccagagtgagttga

gggagcgctcaggtttcccctttcccttccccagagtggcaagggccagatcagacaaat

gagaaccaggctaagcatttcctagcaacctcgcccctcccccaactccatgtgagagcc

ctgctcctccagcattaaccggcctcctgcctgtctttgctaccctgacaccggcctgct

ctgaaggttctctgctggaactctctgggaactggagggcatgctgaggaaggctctata

gcttcctgtcttgtgtctggaagaggaaccttctcttcccaggtcagctgcaagacagac

cacctgctaggccagcccagaagttcagggaggccttggaccccatggctcagatatgcg

gaccagttaacgtgcagggagatcagagcatcttccaaactgggcagaaaaacagcacct

tgtccttcctcagccctgcccaaggcccagcagattccaaaccataacagataaaggcct

agagacacagggaaaggtcctacaagcacctctctgtggagagctctctatgctctggtc

aagtacttggacttcttcccaggaagtgagacctggggtcaggtcacccaaacaagactc

gtgggtgagaggtaatgtcagtgtggctgaccaagaaagggctaaggggactggggagag

agagagagagagagagagagagagagaaggaagaaagagagagagagagaagtaaagaaa

gaaaaagaaaagagagaagagaaaagcaaaaccactcacctggtacacagagtttggagg

gcgttctttaagaatatccctttattccagtgctggggaccagacccaggacaccatcaa

tagatgttaggcaggcactctatcgactaagccatctcccaatctgataaccagtttgac

atcccacctgcctgtcacctttctgaatctgtttcccttgacctagatataggtcaggtg

gctgtgccatggatgaaaacagggccaagttgatttatagtgaagccgttagcttgccag

ccagagctccagctaccaca

**S4. Cloned rat genomic DNA sequence showing the Synapsin I promoter sequence used in transfection studies.** The primer sequences used for cloning (rSYF1 and rSYR1, see Table S1) are highlighted in yellow.

GAAGAGGCTGAATACACATCAGAGTTAGTGCTGCAGGAAATGCTTCTGCATTGCATACCCAGAGTTTCCTTGCTCATCTGAGAGCATGTGTTTTTTCCAGATGTGTGTACTTGTGTGAGATTCTCTGGGTGTGTGTCAATGTGTTGCCTGAACGTGCATTGCTCAATATGCTCATGTGTGTTACCCTGGGCTTGTACATCTACATATATACCTGGATGCCCGTGTGTTCTGTGATGTACATATACCCTGTGTCATTCCTTGTTTTTCTATTTGTGTTATTCCATGTGTTCCTTCAGGCTCTCACTACCCAAGTGTCCACCTCCGCCTGTCTGGTGATGTTTACGCTACCCCGTGCTCTTTTCTTTGCCTGACAGTGTTGTCGTGGAAGACATCTCGCCAGGAACACTGCAGTAAGGAGAATTTCTAGTTTTATGTTCCCCTCCGAGTATGCTTCTATCCCGACCCTCAACCCCAAAATGCCTTCAGAGGTGAAAATCAACACTGGAAACACAAGTATCTGGGAAGGGTAACAATGCAAGTTAGCCTGAGGATTTAGGAGGAGGCTGAAAAACAGAGTAGGAGCCTTACTACGGGTCCAGACCCTACGGACAAGAACCCCCACTCCCACTCCCCAAATTGCGCATTCCCTCCCCCATCAGAGGGGGAGGGGAAGAGGATGCAGCGCGGCGCGGCGCGTGCGCACTGTCGGATTTAGTACCGCGGACAGAGCCTTCGCCCCCGCTGCCGGCGCGCGCCACCACCTCCCCAGCACCAAAGGCGGGCTGACGTCACTCTCCAGCCCTCCCCAAACTCCCCTACCTCACCGCCTTGGTCGCGTCCGTGCAGCGGTGAGTCCAGTCGGGCCGCACCACAAGAGGTGCAAGATAGGGGGGTGCAGGCGCGACCATACGCTCTGCGGCGGCAGAGCCTCAGCGCTGCCTCAGTCTGCAGCGGGCAGCAGAGGAGTCGCGTCGTGCCAGAGAGCGCCGCCGTGCTCCTGAGCCCCTTGCGCTCCGCCCCCGCGGCCCACCGACCCACTGCCCCTT

**Fig. S5.**


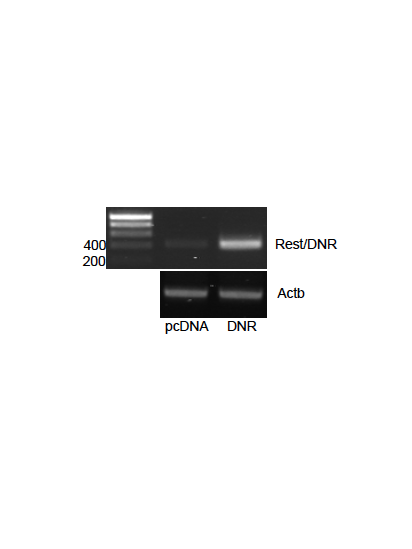


Fig. S5. Over-expression of a dominant-negative REST (DNR) expression construct in HT22 cells. Representative agarose gel electrophoresis image of RT-PCR analysis using primers directed against part of mouse REST (mmRestF1 & mmRestR2; see Table S1) that amplifies a 416bp product from both endogenous Rest mRNA, and the synthetic DNR molecule. Note the high relative product levels in extracts from cells transfected with the DNA construct (DNR) compared with cells transfected with empty pcDNA3.1 (pcDNA). In a separate PCR reaction, the same samples were used for amplification of an *Actb* product, for comparison of transcript levels. Numbers indicate molecular size ladder bands (left) in bp.

**Fig. S6.**

**
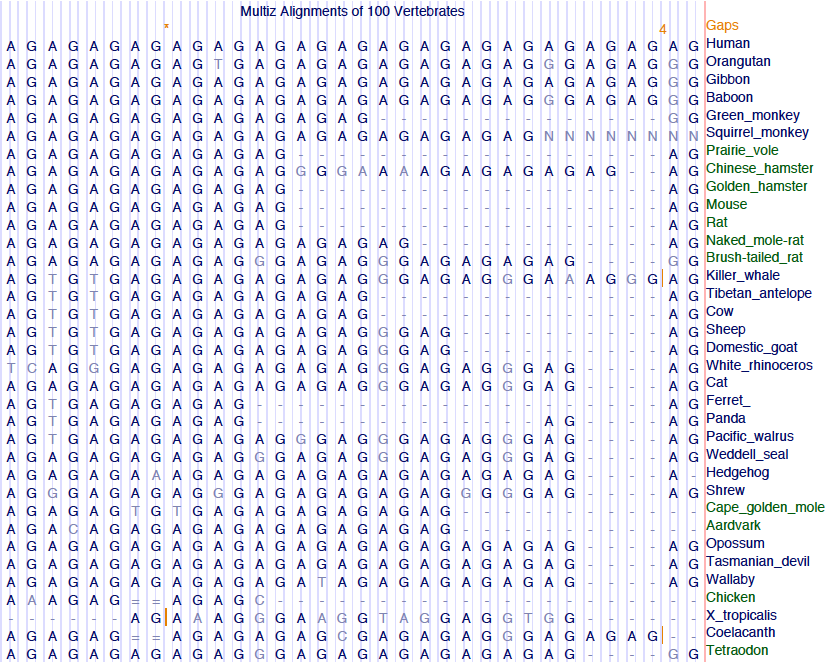
**

**Fig. S6. Conservation of the *Cacng2* GA-repeat sequence across different animal groups.** UCSC browser image showing part of the GA-repeat sequence in the human genome (hg38, chr22:36,703,767-36,703,800) where there is conservation across many species. This region was selected arbitrarily to show a region where at least 5 GA dinucleotide pairs are conserved (5 being the commonly recognized minimum number to constitute a ‘STR’ sequence) Note that at least 5 GA pairs are conserved across many animal groups including different primates, Euarchontoglire**s** including different rodents, *Laurasiatheria* including cow and sheep, *Afrotheria* including aardvark, other mammals including wallaby, *Sarcopterygii* including Coelacanth, and fish, incluidng Tetraodon. Also, notably, analysis of this region (see Figure, above) did not reveal conservation in any birds including chicken, nor in another *Sarcopterygii* representative, *Xenopus sp*. A more extensive phylogenetic analysis would be required to determine the full extent of sequence conservation across the *Cacng2* locus in all database genomes.

**Fig. S7.**

**A.**

**Human**


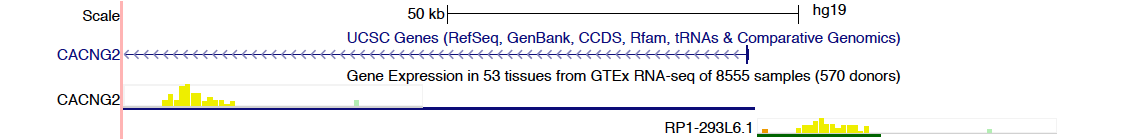


**Mouse**


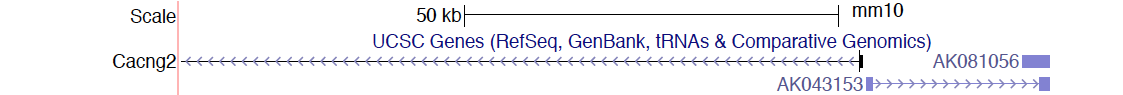


**B.**

**Human**

**
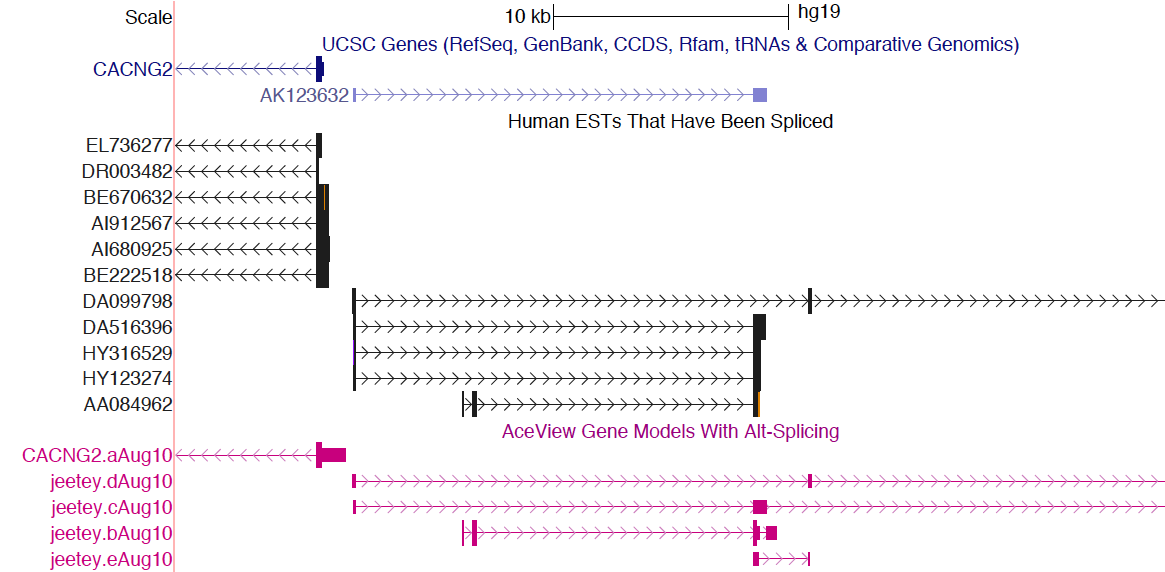
**

**Fig. S7. Bi-directional transcription at the Cacng2 promoter, indicated by annotation of downstream lncRNA sequences. A.** UCSC browser images showing the human (upper, hg19 ) and mouse (lower, mm10) *Cacng2* locus with lncRNA sequences annotated from GTEx (human) and EST (mouse ) analysis. Note that direction of transcription is indicated by arrow direction (mouse AK043153), or text sense (human RP1-293L6.1). **B.** UCSC browser images showing the human (hg19 ) *Cacng2* locus with multiple downstream lncRNA sequences annotated by UCSC (upper, blue), EST analysis (middle, black) and Ace View (lower, pink).

**Fig. S8.**

**
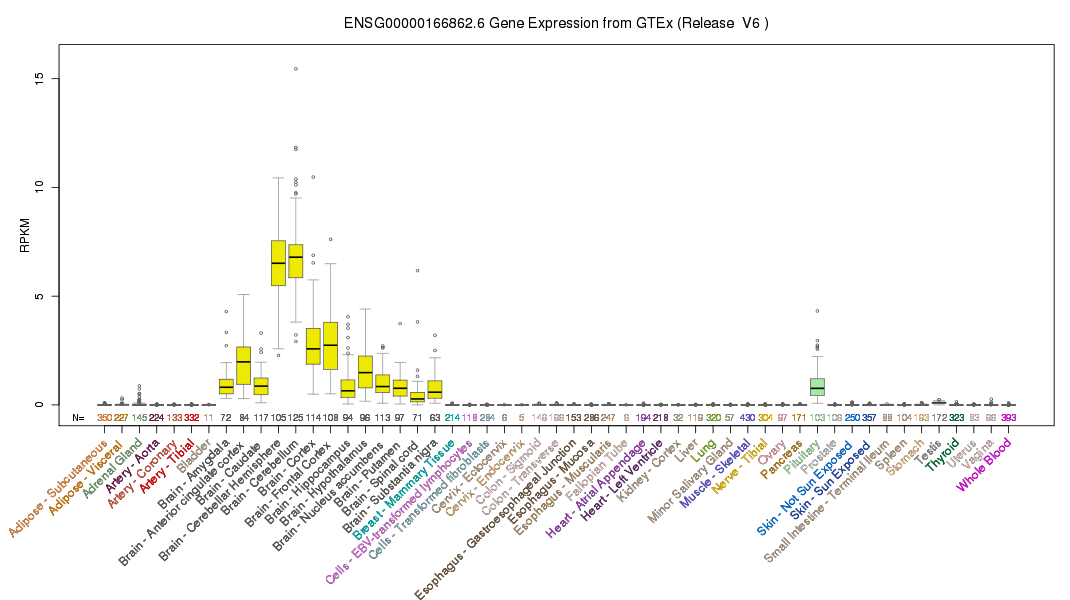
**

**
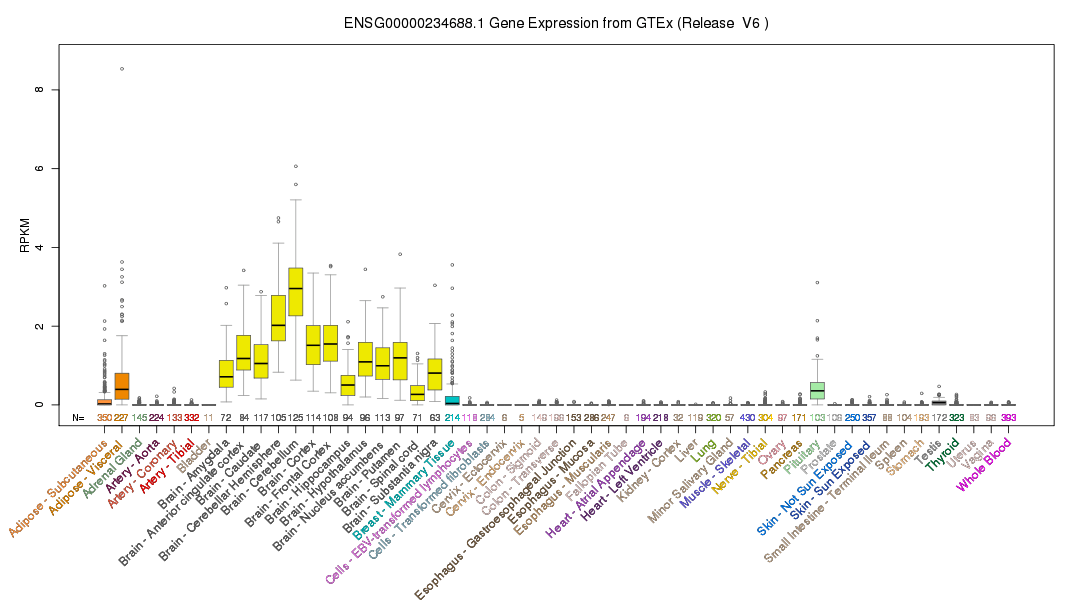
**

**Fig. S8. Similarity of expression profile between human *Cacng2* (upper panel) and the associated lncRNA sequence RP1-293L6.1 (lower panel).** Gtex RNA-seq data across multiple brain regions, and other tissues. Expression levels are expressed as RKPM (reads per kilobase per million mapped reads); note the different X-axis scales on each graph. (GTEx Consortium, 2015).

**S9. Annotated CpG island sequence in the mouse is highly conserved in rat.** UCSC genome sequence derived from the mouse genome (mm10; upper sequence block) was compared with the rat genome using BLAT (lower sequence block). Identical bases are in blue.

>mm10_cpgIslandExt_CpG: 61 range=chr15:78119598-78120185

CGAGCAGCCGCGGTTATTGTTGTTGGCGGCGGGGGTAGTGTCGGCGAAGT GGGGGAGGGAGGGGGTTTCTCCCGGAGAATCGAGGCGGGGTTCCCTCCCC CGATCCGCAAAGCTCCTCGGAAGCCAGCGAGCGAGCGAGCGATGGGGCGC GCTAGCCGGCGTCTTGCAGCAGGCAGGCCCGCCCGCCTCCCCCGCTCACG GCCGCGCAGCCCCGGGCCAGGCGCTCCCAGCTACAGCATCGCCGTGGTGC TGAAACGGACAGCTCACCCCGGCCGGGCTCGTCCACTTACTGCATCGCCG TAGTGCTGAAGTGGACAGCTCCCCACGACCGGGCGCTCTCACCTACAGCA TCGCCGCGGTGCTGGACTGGACAGCTCTCGGAGCAGGAGCGCCCACCTAC TGCGTCGCCGTGGTGCTGAACTGGACAACTCCCCCACTCTGTGCGCTCCC TGCGCTCTGTAGCCGGCCAGGAGTGGGGCCCTGGCCACGGGGCCTCTGTC GTCCGCGGACGGGCCAGGCCAGGGCCAAGCGCAGGGCTGCCTGGCCTCCC GGACTCCTGTTCCACAGTTCCGGAATGAGCGCTGGCCG

Equivalent rat sequence derived from BLAT analysis:

CGAGCAGCCG CGGTTATTGT TGTTGGCGGC GGGGGTAGTG TCGGCGAAGT GGGGGAGGGA GGGGGTTTCT CCCGGAGAAT CGAGGCGGGG TTCCCTCCCC CGATCCGCAA AGCTCCTtGG AAGCCAGCGA GCGAGaGAGC GATGGGGCGC GCTAGCCGGC GTCTTGCAGC AGGCAGGCCC GCCCGCCTCC CCCGCTCgCG GCCGCGCAGC CCCGGGCCAG GCGCTCCCAG CTACAGCATC GCCGTGGTGC TGAAACGGAC AGCTCACCCC GGCCtGGCTC tTCCACTTAC TGCATCGCCG TgGTGCTGAA cTGGACAGCT CCCCgaGACa GGGCGCTCTC ACCTACAGCA TCGCCGCGGT GCTGGACTGG ACAGCTCTCG GAGCAGGAGC GCCCACCTAC TGCGTCGCCG TGGTGCTGAA CTGGACAgCT CCCCCACTCT GTGCGCTCCC aGCGCTCTGT AGCCcGCCAG GAGTGGGGCC gggctACGGG GCCTCTGTtG TCCGCGGACG GGCCAGGCCG GGCTGCCTGG CCTCCCcGAC TCCTGTTCCA AGTTCCGGA ATGAGCGCTG

**S10. Sequences of rat brain lncRNAs deriving from the *Cacng2* locus.** cDNA sequences of the 405bp, 578bp and 491bp PCR products amplified from rat brain. Primer sequences (see Table S1) are highlighted in yellow. Sequences in blue are regions of identity derived from BLAT analysis of the rat genome, showing blocks of individual exon sequence with their respective locations in the genome sequence, and exon-flanking sequence in lower-case black font. Single bases highlighted in purple are divergent from the database genome sequence (Rn6). Numbers below each exon where appropriate are exon size (bp). Note that the GA (CT/TC) repeat sequence length in the 491bp lncRNA product is characteristic of SD rat, as compared with the database BN rat.

405bp (3-exon) product

TGGTGTTTCTTCCAGATCAGCCTCCTTTTCATTCCAACTCTACGTGCCCAGAGCTTCAAATACAGCCCTCCCATCACAGTCAGACTCTCACAAAACTTCAATCAGCTGCTTCTCCTCCTGGAAGAGAACCTGGATGGGAGGAAGCTGGAAGGTGGGAAAGAAGAGGTGTGGAGTCAGCTGCATCTGGCCTGGATTCCAGTTCTGCTGTCTATTTGCTGTGTGGTCCCATGCAGGGATTTGACTTCTCTGGGCTCAGCCTCATCTCAGAACTCAAGGCACCCGAGATGAAAGGCAGAGAAACAGCAGAAACAAAAGCTCCGGTCTTGAGCTTTGCCGCCTTCCCATCCCCCTCACCCCTCACCCCTCACCAGCACTTCCTCGAGTCCTGAAAGTCTGCTTCGGTCT

TGGTGTTTCT TCCAGATCAG CCTCCTTTTC ATTCCAACTC TACGTGCCCA 119353440 GAGCTTCAAA TACAGCCCTC CCATCACAGT CAGACTCTCA CAAAACTTCA 119353490 ATCAGCTGCT TCTCCTCCTG GAAGAGAACC TGGATGGgta attacaaaca 119353540

137

ctttattgtt ttgaaccagG AGGAAGCTGG AAGGTGGGAA AGAAGAGGTG 119357940 TGGAGTCAGC TGCATCTGGC CTGGATTCCA GTTCTGCTGT CTATTTGCTG 119357990 TGTGGTCCCA TGCAGGGATT TGACTTCTCT GGGCTCAGCC TCATCTgtaa 119358040

127

tttgatcatt tatttatact ctattttttt cagCAGAACT CAAGGCACCC 119376140 GAGATGAAAG GCAGAGAAAC AGCAGAAACA AAAGCTCCGG TCTTGAGCTT 119376190 TGCCGCCTTC CCATCCCCCT CACCCCTCAC CCCTCACCAG CACTTCCTCG 119376240 AGTCCTGAAA GTCTGCTTCG GTCTgtcttc ggagtcaagg tgctggggtc 119376290

141

578bp (4-exon) product

TGGTGTTTCTTCCAGATCAGCCTCCTTTTCATTCCAACTCTACGTGCCCAGAGCTTCAAATACAGCCCTCCCATCACAGTCAGACTCTCACAAAACTTCAATCAGCTGCTTCTCCTCCTGGAAGAGAACCTGGATGGGAGGAAGCTGGAAGGTGGGAAAGAAGAGGTGTGGAGTCAGCTGCATCTGGCCTGGATTCCAGTTCTGCTGTCTATTTGCTGTGTGGTCCCATGCAGGGATTTGACTTCTCTGGGCTCAGCCTCATCTGTAATTTGGACCCCGTGGTGAGAGAGTAGTGATTGCAGATGGGGCATTCACGGCCTCTCCCCACTGTTCTCGAGCTCATTC

TGAGGACTAATGCCACGCATGCGGCTGACCCAGCTTTTCAGACACGTGACCAAGGTTTTGGAGTACCTACGAGGCTCGATCTAGCGTCTTCCCTGAACTCAAGGCACCCGAGATGAAAGGCAGAGAAACAGCAGAAACAAAAGCTCCGGTCTTGAGCTTTGCCGCCTTCCCATCCCCCTCACCCCTCACCCCTCACCAGCACTTCCTCGAGTCCTGAAAGTCTGCTTCGGTCT

TGGTGTTTCT TCCAGATCAG CCTCCTTTTC ATTCCAACTC TACGTGCCCA 119353440 GAGCTTCAAA TACAGCCCTC CCATCACAGT CAGACTCTCA CAAAACTTCA 119353490 ATCAGCTGCT TCTCCTCCTG GAAGAGAACC TGGATGGgta attacaaaca 119353540

137

ctttattgtt ttgaaccagG AGGAAGCTGG AAGGTGGGAA AGAAGAGGTG 119357940 TGGAGTCAGC TGCATCTGGC CTGGATTCCA GTTCTGCTGT CTATTTGCTG 119357990 TGTGGTCCCA TGCAGGGATT TGACTTCTCT GGGCTCAGCC TCATCTgtaa 119358040

127

accaaccctt ctctccttcg gctgtttcac cagGTAATTT GGACCCCGTG 119361640 GTGAGAGAGT AGTGATTGCA GATGGGGCAT TCACGGCCTC TCCCCACTGT 119361690 TCTCGAGCTC ATTCTGAGGA CTAATGCCAC GCATGCGGCT GACtCAGCTT 119361740 TTCAGACACG TGACCAAGGT TTTGGAGTAC CTACGAGGCT CGATCTAGCG 119361790 TCTTCCCTGg tatctactgg gttcaataag aaataaatgg aggtgggtgg 119361840

176

tttgatcatt tatttatact ctattttttt cagcagAACT CAAGGCACCC 119376140 GAGATGAAAG GCAGAGAAAC AGCAGAAACA AAAGCTCCGG TCTTGAGCTT 119376190 TGCCGCCTTC CCATCCCCCT CACCCCTCAC CCCTCACCAG CACTTCCTCG 119376240 AGTCCTGAAA GTCTGCTTCG GTCTgtcttc ggagtcaagg tgctggggtc 119376290

138

491bp (1-exon) product

ATCGCCGTGGTGCTGAAACGGACAGCTCACCCCGGCCTGGCTCTTCCACTTACTGCATCGCTGTGGTGCTGAACTGGACAGCTCCCCGAGACAGGGCGCTCTCACCTACAGCATCGCCGCGGTGCTGGACTGGACAGCTCTCGGAGCAGGAGCGCCCACCTACTGCGTCGCCGTGGTGCTGAACTGGACAGCTCCCCCACTCTGTGCGCTCCCAGCGCTCTGTAGCCCGCCAGGAGTGGGGCCGGGCTACGGGGCCTCTGTTGTCCGCGGACGGGCCAGGCCGGGCTGCCTGGCCTCCCCGACTCCTGTTCCACAGTTCCGGAATGAGCGCTGTCCAGGGCCTCCTGGAGCTGGGAGGAAGGCGTAGCTGGCGATCTCTCTCTCTCTCTCTCTCTCTCTCTCTCTCTCTCTCTCTCTCTCTCTCTCCACTTCCCTCCCCCCTTGGTGTTTCTTCCAGATCAGCCTCCTTTTCATTCCAACTCTACGTGCCC

ATCGCCGTGG TGCTGAAACG GACAGCTCAC CCCGGCCTGG CTCTTCCACT 119352996 TACTGCATCG CcGTGGTGCT GAACTGGACA GCTCCCCGAG ACAGGGCGCT 119353046 CTCACCTACA GCATCGCCGC GGTGCTGGAC TGGACAGCTC TCGGAGCAGG 119353096 AGCGCCCACC TACTGCGTCG CCGTGGTGCT GAACTGGACA GCTCCCCCAC 119353146 TCTGTGCGCT CCCAGCGCTC TGTAGCCCGC CAGGAGTGGG GCCGGGCTAC 119353196 GGGGCCTCTG TTGTCCGCGG ACGGGCCAGG CCGGGCTGCC TGGCCTCCCC 119353246 GACTCCTGTT CCACAGTTCC GGAATGAGCG CTGTCCAGGG CCTCCTGGAG 119353296 CTGGGAGGAA GGCGTAGCTG GCGATCTCTC TCTCTCTCTC TCTCTCTCTC 119353346 TCTCTCTCTC TCTCTCTCTC TCTCTCtcCA CTTCCCTCCC CCCTTGGTGT 119353396 TTCTTCCAGA TCAGCCTCCT TTTCATTCCA ACTCTACGTG CCCagagctt 119353446

**Fig. S11.**

**Fig.S11.** Tissue-specific expression of 5’ lncRNA sequence associated with the *Cacng2* promoter. Representative image of agarose gel electrophoresis analysis of PCR-amplified lncRNA and *Actb* mRNA from different tissues showing brain-specific expression of a 491bp product. Numbers on the left are sizes in base pairs.
